# Supplementary material for: A Novel Gammapartitivirus That Causes Changes in Fungal Development and Multi-Stress Tolerance to Important Medicinal Fungus Cordyceps chanhua
Source: J Fungi (Basel). 2022 Dec 16;8(12):1309. doi: 10.3390/jof8121309 (PMC9782574; doi:10.3390/jof8121309)
Supplement: Supplementary file 1 [file jof-08-01309-s001.zip › 1.Supplementary Figures S1.pdf]

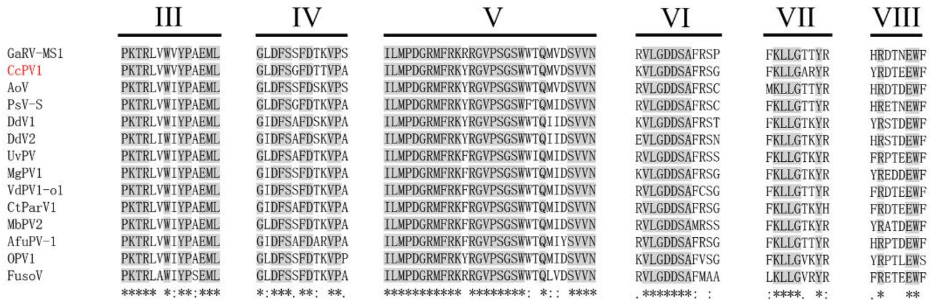

Figures S1: Alignment of RdRp sequences between CcPV1 and other members of *Partitiviridae*. CcPV1 (Cordyceps chanhua partitivirus 1), GaRV-MS1 (Gremmeniella abietina RNA virus; MS1AAM12240.1), AoV (Aspergillus ochraceous virus; ABV30675.1), PsV-S (Penicillium stoloniferum virus S; YP_052856.2), DdV1 (Discula destructiva virus 1; AAG59816.1), DdV2 (Discula destructiva virus 2; AAK59379.1), UvPV (Ustilaginoidea virens partitivirus; AGO04402.1), MgPV (Magnaporthe grisea partitivirus 1; AZT88596.1), VdPV1-o1 (Verticillium dahliae partitivirus 1; YP_009164038.1), CtParV1 (Colletotrichum truncatum partitivirus 1; ALF46547.1), MbPV2 (Metarhizium brunneum partitivirus 2), AfuPV-1 (Aspergillus fumigatus partitivirus 1; CAY25801.2), OPV1 (Ophiostoma partitivirus 1; CAJ31886.1), FusoV (Fusarium solani virus 1; BAA09520.1)
