# Supplementary material for: A Novel Gammapartitivirus That Causes Changes in Fungal Development and Multi-Stress Tolerance to Important Medicinal Fungus Cordyceps chanhua
Source: J Fungi (Basel). 2022 Dec 16;8(12):1309. doi: 10.3390/jof8121309 (PMC9782574; doi:10.3390/jof8121309)
Supplement: Supplementary file 1 [file jof-08-01309-s001.zip › 1.Supplementary Figures S2.pdf]

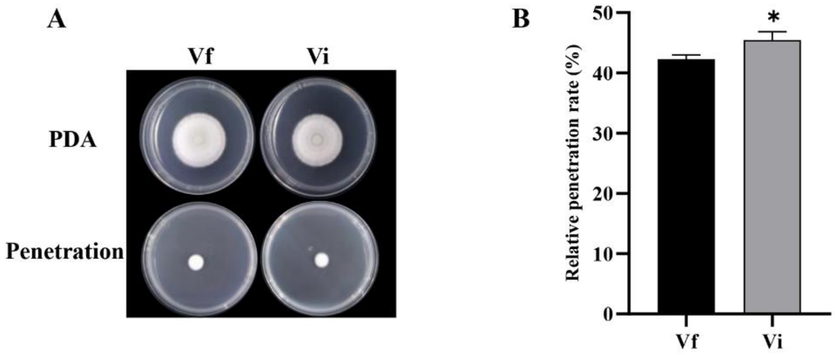

Figure S2. Penetration determination of Vf and Vi strains. (A) Colony morphology of Vf and Vi strains on PDA and after removing cicada wings. (B) Relative penetration rate of strains Vf and Vi growting on PDA affixed with cicada wings. * $p < 0.05$.
